# Facilitating advance care planning in oncology: study protocol for the evaluation of a tailored implementation of an advance care planning tool (the INDEED study)

**DOI:** 10.1186/s12904-026-02285-9

**Published:** 2026-08-14

**Authors:** Joica A. M. Benschop, Ida J. Korfage, Agnes van der Heide, Annemieke van der Padt-Pruijsten, Carin C. D. van der Rijt, Wendy H. Oldenmenger

**Affiliations:** 1 Department of Medical Oncology, Erasmus MC Cancer Institute, University Medical Centre Rotterdam, P.O. Box 2040, Rotterdam, 3000 CA the Netherlands; 2 Department of Public Health, Erasmus MC, University Medical Centre Rotterdam, Rotterdam, the Netherlands; 3 Department of Internal Medicine, Maasstad Hospital, Rotterdam, the Netherlands

**Keywords:** Advance care planning, Implementation, Tailored strategies, Hospital, Oncology, Palliative and supportive care, Electronic health care record, Mixed methods study.

## Abstract

**Background:**

Advance care planning (ACP) improves patient outcomes, but integration of ACP in daily practice for patients with cancer in hospitals remains suboptimal. Systematic implementation of tools to facilitate ACP could overcome the barriers for ACP. In this study we will evaluate the multifaceted tailored implementation of a digital ACP tool embedded in the electronic health record (EHR) in daily clinical cancer care. We will analyse the implementation process, its effect on use of the ACP tool, and the impact of use of the tool on healthcare use and healthcare costs.

**Methods:**

We will perform a multicentre, convergent mixed-methods implementation study. The digital ACP tool supports healthcare professionals in conducting and documenting ACP conversations, and sharing this documentation with colleagues within and outside the hospital. The implementation will encompass four phases guided by the Implementation of Change model: 1) diagnostic analysis; 2) tailored implementation protocol development; 3) protocol implementation and testing; and 4) evaluation of the implementation process and its effects. We will evaluate both qualitative and quantitative aspects of the implementation process. Additionally, use of the tool and its impact on in-hospital healthcare use in the last three months of life and associated costs are measured.

**Discussion:**

This study will provide knowledge on how to effectively implement ACP in routine care in hospitals for patients with cancer, by identifying effective implementation strategies. Furthermore, it will give insight in the effect of integration of ACP conversations in daily clinical care on healthcare use of patients with cancer in the last three months of life and the associated costs.

**Trial registration:**

This study is registered at ClinicalTrials.gov (NCT06350968, Registration date: April 8, 2024).

**Supplementary Information:**

The online version contains supplementary material available at https://doi.org/10.1186/s12904-026-02285-9.

## Background

Advance care planning (ACP) originally entailed specific decisions about medical treatment at the end of life. However, ACP has developed into a process encompassing relational, emotional, and social factors, aiming to better align treatment with patients’ needs, hopes and preferences. ACP is a process of “enabling individuals to define goals and preferences for future medical treatment and care, to discuss these goals and preferences with family and healthcare professionals, and to record and review these preferences if appropriate” [1]. Hence, ACP is considered relevant for many patients, irrespective of age, stage of illness or prognosis. For patients with cancer in the palliative phase, ACP is regarded an integral component of care [2].

Results from previous studies on the effects of ACP on patient and healthcare outcomes are promising. For instance, several studies demonstrated that ACP improves the quality of patient– clinician communication, increases patient satisfaction and quality of life, as well as the use of palliative care, while reducing unwanted admissions to the hospital and healthcare costs [3, 4]. However, 30–38% of patients with cancer at the end of life (EoL) do not engage in conversations about EoL-care with their physician [5–7]. Furthermore, research in oncology indicated that the rates of documented ACP conversations remain low, and when ACP is documented, it occurs often late in the treatment trajectory [8–12]. Consequently, patients’ goals and preferences frequently remain unknown and inaccessible, thereby hindering effective team communication regarding treatment and care.

Barriers for healthcare professionals to have ACP conversations include time constraints, lack of training in communication about serious illness, discomfort talking about EoL-issues, and fragmented medical record services [13, 14]. ACP tools or templates for documenting patients’ goals and preferences, which are embedded in the electronic health record (EHR), could support the process of ACP, particularly for non-palliative care professionals. Moreover, such tools facilitate the exchange of ACP information both within and outside the hospital, making it readily accessible to all healthcare professionals involved in the patient’s care. However, use of such tools in daily clinical practices by healthcare professionals requires systematic implementation [15]. Systematic implementation of the ACP tool, appropriately tailored to the context and aligned with the local barriers, is essential for the successful integration of ACP into daily clinical practice. Therefore, we will perform a study to evaluate the multifaceted tailored implementation of a digital ACP tool in nine hospitals in the Netherlands. In this paper we describe the design of our study.

## Methods

### Aim

In this study we will evaluate the multifaceted tailored implementation of a digital ACP tool embedded in the electronic health record (EHR) in daily clinical cancer care. We will analyse the implementation process, its effect on use of the digital ACP tool, and the impact of use of the tool on healthcare use and healthcare costs.

### Design

We will conduct a multicentre, convergent mixed-methods implementation study. The digital ACP tool was originally expected to be implemented between October 2023 and June 2026. However, due to insurmountable delays during the implementation process, the study period has been extended to December 2026. The tool will be implemented in three clusters using a stepped-wedge design. Each cluster will comprise two to four hospitals, depending on their readiness, local approval procedures, and availability of healthcare professionals to participate in the implementation. Each hospital will tailor the implementation to its own context and setting, following the Implementation of Change model of Grol & Wensing [15].

### Setting

The ACP tool will be implemented and evaluated in nine hospitals in the south-west region of the Netherlands: one academic hospital, seven general hospitals, and one hospital outpatient clinic. The hospitals collaborate in the cancer network Concord and patients are often treated in multiple hospitals within the network.

### Study population

The study population includes patients and healthcare professionals, i.e. oncologists, haematologists, nurse practitioners, nurses, and social workers. Study outcomes will be examined for patients aged ≥ 18 years, who were treated in one of the participating hospitals for a haematological or solid tumour, and who deceased during the study period. No exclusion criteria were applied for participating in this study.

### The digital ACP tool

All participating hospitals use the EHR system HiX from Chipsoft^®^. HiX embedded an ACP tool. This ACP tool was developed in accordance with a nationally agreed set of core indicators for palliative care [16] and with healthcare professionals from 20 Dutch hospitals. The tool includes generally recommended components such as patients’ goals and values, prognosis, treatment preferences, and decision-making about medical, nursing, and social care. All topics of the ACP tool are outlined in Table 1. A more detailed explanation of all topics of the tool can be found elsewhere [9]. Despite its theory- and practice-based design, there has been low engagement with its use.

**Table 1:** Topics of the ACP tool

• Social context (e.g., living situation, marital status, profession, hobbies)
• Meaning (e.g., what is important to the patient, concerns)
• Organization of care (e.g., already involved caregivers, patient preferred place of care and death)
• Decision-making / advance directive (e.g., whether the patient wants to be readmitted to hospital, living will)
• Questionnaires (e.g., HADS, Distress Thermometer)
• Communication (e.g., how the patient prefers to be approached)
• Physical status (e.g., consciousness, WHO-performance status, symptoms)
• Psychological status (e.g., illness understanding and emotions)
• Life expectancy
• Prompts for palliative and supportive care consultation (e.g., surprise question)
• Follow-up after death (with relatives)

Healthcare professionals are able to record and review ACP conversations within the tool, and update them as necessary. Patients can be given an active role in communicating and documenting their preferences by sending them an electronic questionnaire. Patients’ responses are directly accessible in the ACP tool. The tool can be accessed via a button at the front page of the EHR. Documentation can be shared with healthcare professionals outside the hospital digitally, or on paper.

### Implementation procedure

**Fig. 1:**
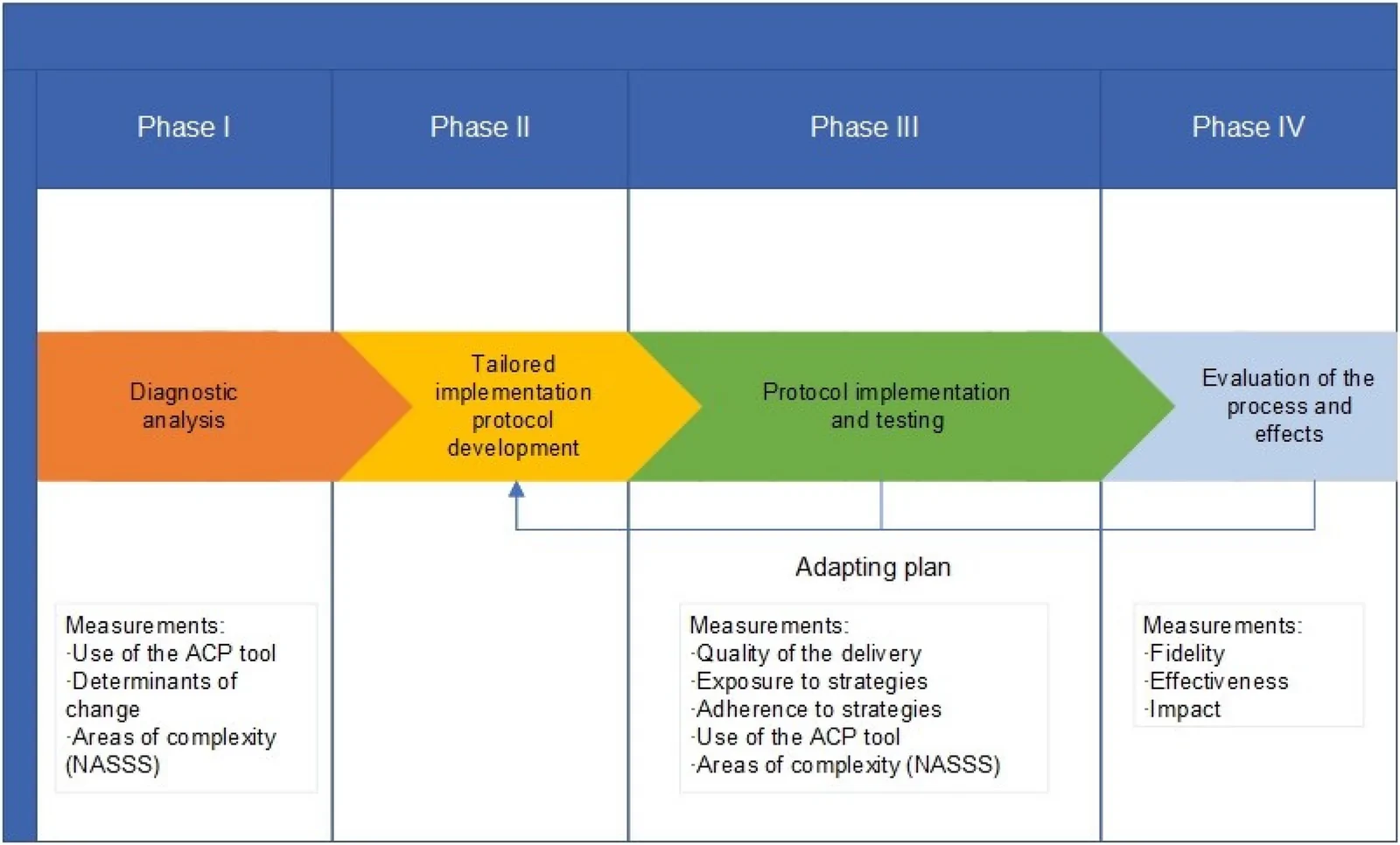
Four phases of ACP tool implementation guided by Grol & Wensing [15]

Each hospital completes the implementation of the ACP tool tailored to its own context and setting, following the four phases as described in Fig. 1. It is estimated that the implementation process will take 9 months per hospital.

#### Phase I – diagnostic analysis

The actual use of the ACP tool at baseline will be measured. Additionally, interviews with patients and healthcare professionals will be conducted to reveal determinants of current practices, opinions, attitudes, and beliefs regarding ACP and the use of the tool. Subsequently, the complexity of the implementation will be mapped using the NASSS (non-adoption, abandonment, or fail to scale-up, spread and sustain over time) framework [17]. The NASSS framework is particularly useful for eHealth implementations like this ACP tool. It enables a systematic approach to explore the complexities of the ACP tool in relation to its implementation: the more complex, the more risk of uptake failure. NASSS focuses on seven key domains; Condition, Technology, Value proposition, Adopters, Organization, External context, and Embedding and Adaptation over time. Repeated use of the framework during the implementation period will help adapting the implementation protocol if necessary.

#### Phase II – tailored implementation protocol development

Each participating centre will establish a dedicated team including a local project leader, healthcare professionals, patients, and other key stakeholders involved in cancer care. A researcher will participate in all teams. First, the teams will choose tailored implementation strategies and outcomes for different stakeholder groups based on the complexities encountered in Phase I. The strategies are aimed at improving the use of the ACP tool. Implementation strategies will be selected from the Expert Recommendations for Implementing Change (ERIC) taxonomy [18]. The strategies focus at different levels: organizational, departmental, and individual. Second, the strategies will be further defined as concrete activities for each stakeholder group. Finally, the team will also define hospital-specific fidelity targets, i.e. the extent to which the stakeholder groups are exposed to and adhere to implementation activities, and an ACP tool usage target, i.e. the extent to which the ACP tool is used in clinical practice. Strategies, activities, and targets will be described in a multifaceted tailored implementation protocol, which can be finetuned during the implementation.

#### Phase III – protocol implementation and testing

The stakeholder groups identified in Phase II will be exposed to implementation activities as defined in the protocol. During this period, the team will meet frequently to evaluate the progress and adapt the protocol in case fidelity targets are not met, or when the use of the tool does not improve. The NASSS toolkit [19] will offer suggestions for improvement in such instances.

#### Phase IV – evaluation of the process and effects

A final evaluation of the implementation process and outcomes will be conducted using the measurements as described below.

### Study measurements

**Fig. 2:**
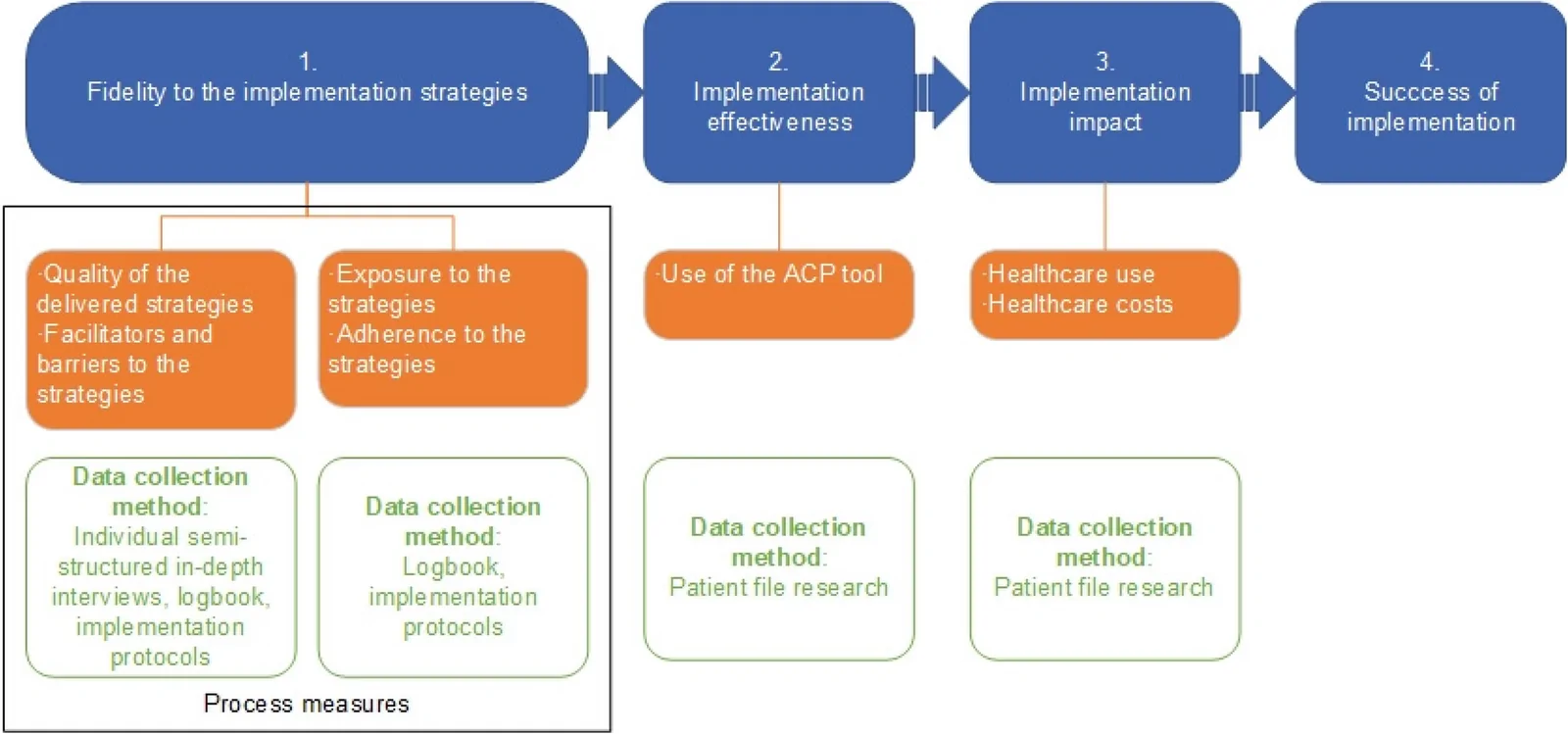
Outcomes of implementation categorized according to Proctor et al. [20]

The implementation will be evaluated following Proctor’s Conceptual Framework for Implementation Outcomes (IOF) [20]. Outcomes will be analysed for each hospital separately and in an overall analysis. Figure 2 illustrates the measurements and data collection methods.

### Fidelity to the implementation strategies

The fidelity to the implementation strategies will be measured both qualitatively and quantitatively.

#### Qualitative measurements and analysis

In each hospital, we will conduct semi-structured, in-depth interviews with purposively selected patients and five to ten healthcare professionals from the stakeholder groups as defined in Phase II. An interview guide was developed based on the NASSS CAT-LONG interview guide (Supplementary File A) [21]. Interviews will be audiotaped, transcribed verbatim, and pseudonymized for analysis. Additionally, the implementation protocols and the logbook with field notes from the researcher will be used for analysis.

All data from the interviews, protocols, and the logbook will be analysed inductively and deductively at several levels as an iterative process of constant comparison based on thematic analysis [22]. This analysis will identify experiences, underlying motivations and behaviours. Interviews will be coded using NVivo (version 12 for Windows). The data will be structured by thematic codes, derived from relevant literature and the NASSS framework [17]. Besides, the implementation protocols and textual field notes will be discussed by the research team, looking for patterns, reflecting, and giving meaning to the notes.

#### Quantitative measurements and analysis

Quantitative data on fidelity to the implementation strategies will be collected per hospital. Exposure and adherence rates for the different stakeholder groups will be recorded in the logbook of the researcher. For example, when the implementation strategy ‘train and educate stakeholders’ is chosen by the dedicated team, and the corresponding implementation activity is ‘training in the use of the digital ACP tool for nurses’, the quantitative fidelity measure will be the number of nurses that attended the training relative to the total number of nurses in the stakeholder group. No demographic data of participants will be collected. Outcomes on fidelity measurements will be presented using descriptive statistics.

### Implementation effectiveness

Implementation effectiveness is operationalized as the use of the ACP tool. Usage will be measured by collecting data from the EHR files of all patients with a haematological or solid tumour, aged ≥ 18 years, who were treated in one of the participating hospitals, and who deceased during study period. Data will be collected 3 to 0 months before, 9 to 12 months after, and 19 to 21 months after start of the implementation period in each hospital. Use of the tool will be calculated as the percentage of patients for whom the ACP tool was ‘activated’. The ACP tool is considered to have been activated when at least one item in the tool has been completed. Additionally, more detailed information on the extent to which topics of the ACP tool are completed will be assessed, as well as the time before death in which the tool was activated.

### Implementation impact

The impact of the implementation aiming to promote the use of the ACP tool is assessed through in-hospital healthcare use and its associated costs. Pre- and post-implementation, healthcare use in the last 90 days of life of the study population will be measured. We have chosen a set of indicators for healthcare use, based on literature and insights from clinical experts [23, 24]. Consequently, healthcare use is operationalized by the number of Emergency Department (ED) admissions, Intensive Care Unit (ICU) admissions, surgeries, hospital admissions, and anti-tumour therapies. Additionally, we investigate how often a team of palliative care experts is involved in the treatment. These indicators will be collected through EHR file research. Subsequently, the indicators will be translated to Dutch national defined costs [25] and reported in Euros (€).

### Sample size

Since this study is an implementation study, no formal sample size calculation is required. Nevertheless, we did conduct a sample size calculation for an important patient-related outcome: in-hospital healthcare use and associated costs. Before the introduction of the ACP tool, 40% of patients still received medical interventions in the last three months of their lives [7]. To detect a 30% reduction with 80% power and a two-sided alpha of 5%, a total of 700 patients is required: 350 with an active ACP tool and 350 without an active ACP tool. These numbers are feasible within the Concord network of nine participating hospitals.

### Data analysis

Data on use of the ACP tool and the different indicators for in-hospital healthcare use will be presented using descriptive statistics. The Chi-square ($\:{X}^{2}$) test will be used to compare use of the ACP tool pre- and postimplementation. Furthermore, the Chi-square ($\:{X}^{2}$) test will be used to examine the difference between the individual indicators for in-hospital healthcare use pre- and postimplementation. In both tests, statistical significance is defined as *P* < .05. All quantitative data will be analysed using SPSS Statistics for Windows, version 32.0.0 (SPSS Inc, Chicago, IL) [26].

For the overall analysis, we will first assess whether hospitals meet their pre-defined target for the use of the digital ACP tool. Second, qualitative and quantitative data analyses regarding the fidelity of the implementation strategies will be integrated [27]. We will then assess whether and how the use of the ACP tool is impacted by the fidelity to the implementation strategies. Further, the impact of the use of the ACP tool on healthcare use and associated healthcare costs after implementation will be determined.

## Discussion

In the INDEED study, we will evaluate the systematic, multifaceted, tailored implementation of a digital ACP tool in daily clinical practice in a large-scale study design, and measure its impact on healthcare use among patients with cancer in the last phase of life, as well as the associated costs. We anticipate that the evaluation and thorough documentation of the implementation strategies and outcomes will identify effective approaches for sustainable integration of ACP in cancer care.

Previous studies have primarily focused on evaluating the effectiveness of ACP interventions [28]. They focused less on the implementation of these interventions in clinical practice. In our study, we will describe the systematic implementation of an ACP tool in clinical cancer care, following implementation theories, models and frameworks (TMFs) [15, 18, 20, 29]. Guided by these TMFs, we are able to consider the context in which the ACP tool will be implemented. Purposefully tailoring the implementation strategies to the contexts of the individual hospitals will provide us with knowledge on the effectiveness of strategies for implementing ACP in hospital settings.

The aim of the implementation is to improve ACP conversations between healthcare professionals and patients, as well as their documentation in an accessible tool. Determining an overall usage target for documentation within the ACP tool for this study is challenging. In other studies, we found documentation outcomes ranging from 54% to 96% [8–10, 12, 30]. However, these studies used various definitions of ‘documentation’, ranging from documentation in tools or templates to progress notes or the rate of palliative care consultations. Additionally, the hospitals in our study have different baseline usage rates. For that reason, we decided not to focus on an overall usage rate, but to set targets for each hospital separately.

Given that we are using anonymous data on an aggregated level for analyses in this study, we can provide an overall analysis of use of the ACP tool for each hospital. The results will provide insight into the extent of ACP documentation within the tool in general, i.e. the number of ‘active’ ACP tools within the study population, but will not offer information regarding the quality of the conversation. However, we will provide insight into which topics of the tool have been completed and the number of days before death on which the tool was activated. Furthermore, the ACP tool implemented in this study does not provide a specific location for documenting that a patient refused an ACP conversation. Therefore, we expect that the healthcare professionals will document this is in progress notes outside the ACP tool. This may have an effect on data on the use of the tool. Unfortunately, we are not able to test this hypothesis, because we will use data on an aggregated level.

We expect to encounter a challenge in the INDEED study. Adequate implementation of the ACP tool is time-consuming and pressure on healthcare professionals is high. Therefore, it is important to minimize the burden of implementation. To reduce this burden, we will involve experienced local project managers who are in close contact with the healthcare professionals who will function as implementation leaders.

We are able to perform limited quantitative analyses because we will use anonymized aggregated data and population-based quality indicators [23, 24], supplemented with insights from clinical experts, to analyse healthcare use. With this data, we are not able to analyse these indicators as potential inappropriate care for individual patients; clinical factors may justify an acute care intervention and personal preferences may differ.

Furthermore, we will not be able to accurately measure the extent to which documented ACP information is shared with colleagues outside the hospital, such as general practitioners, nursing homes, or hospices. We recognize that collaboration with out-of-hospital healthcare professionals is important in ACP for patients with cancer and contributes to a reduction in administrative burden. However, analysing exchange of ACP information of patients requires a more comprehensive analysis.

In conclusion, this will be one of the first studies that identifies effective approaches for sustainable integration of ACP in clinical cancer care, which can be utilized for further implementation.

## Supplementary Information

Supplementary Material 1.

## Data Availability

No datasets were generated or analysed during the current study.

## Abbreviations

ACP: Advance care planning
EoL: End of Life
EHR: Electronic health record
HADS: Hospital Anxiety and Depression Scale
WHO: World Health Organization
NASSS: Non-adoption, abandonment, or fail to scale-up, spread and sustain over time
ERIC: The Expert Recommendations for Implementing Change
ED: Emergency Department
ICU: Intensive Care Unit
TMFs: Theories, models and frameworks
