# Supplementary material for: Facilitating advance care planning in oncology: study protocol for the evaluation of a tailored implementation of an advance care planning tool (the INDEED study)
Source: BMC Palliat Care. 2026 Aug 14;25:323. doi: 10.1186/s12904-026-02285-9 (PMC13695286; doi:10.1186/s12904-026-02285-9)
Supplement: Supplementary file 1 [file 12904_2026_2285_MOESM1_ESM.docx]

Supplementary file A. Interview guide for healthcare professionals based on NASSS-CAT Long interview guide and relevant literature

**FRONT PAGE**

| **Project objective** | The aim of this project is to implement an advance care planning (ACP) documentation tool into (haemato-)oncological care in hospitals collaborating in the Concord cancer network. The implementation must ensure that personal preferences and agreed treatment decisions result in goal concordant care. This research project investigates effective strategies for implementation of ACP in oncological care and will develop an implementation plan for national rollout across all palliative care consortia. |
| --- | --- |
| **Main research question** | What are effective strategies for implementation of ACP into daily care for (haemato-)oncological patients within the Concord cancer network, to ensure integration of advance care planning early in the disease trajectory? |
| **Definition of advance care planning (ACP)** | ACP is a process of “enabling individuals to define goals and preferences for future medical treatment and care, to discuss these goals and preferences with family and healthcare professionals, and to record and review these preferences if appropriate”(1) |
| **Target group of this interview** | Healthcare professionals |
| **Assumptions** | Healthcare professionals working in (haemato-)oncology are expected to be familiar with the concept of ACP, as it is a popular research topic and included in national policies. However, there have been a lack of implementation so far, so not everyone may be using the ACP documentation tool embedded in the electronic health record in their daily practice. |
| **Required information** | Age  Occupation and hospital  # Years of work experience |

**INTERVIEW**

| **Introduction** | - Welcome  - Introduction  - Thank you for your time  - This interview will last approximately 30-45 minutes  - Is it okay if we use informal address (first names)?  - The reason I’d like to speak with you today is to understand to what extent you are currently using the ACP documentation tool and what might helped or prevented you to do so? - I will record the conversation and treat the recording confidentially. - Do you have any questions before we begin? |
| --- | --- |
| **Opening questions** | **Topics/follow up questions** |
| To what extent is it normal/customary for you to ask your patients what is important to them in life and what goals they still wish to achieve? | - Why or why not? |
| If yes, how is that for you to ask these questions? | - Difficult/easy |
| If no, do you think healthcare providers should be aware of this information of their patients? | Why?  - Essential information for treatment decisions  - Alignment with values  - Healthcare professional-patient relation  - Talking about death and dying  - Social pressure |
| Who do you think who is responsible to discuss these topics? | - Primary physician - General practitioner - Patient - Nurse - Allied health professionals - Collaboration with colleagues |
| How do you discuss these topics with your patient? | - Preparing patients  - Who should take the initiative to start? |
| What exactly do you discuss with them? | - What is important in life - What the patient wants you to do for them - Living situation and hobbies - Euthanasia/palliative sedation - Pain management - Medical care in the final phase of life - Concerns about loved ones - Way of saying goodbye and funeral wishes |
| With which patients do you have these conversations? | - Characteristics of those patients  - Condition or illness - Young/old - Curative/palliative treatment - Ethnicity |
| Do you involve the patient’s loved ones? | - How do you do that?  - Why do you find that important? |
| When do you start such conversations? | - Immediately after diagnosis - After marking the palliative phase - Change in illness - When the patient indicates |
| What is the value of these conversations for you? | - What is its value? - What does it contribute to? |
| Does it happen that patients’ wishes are unknown or not discussed? Why is that? | -Lack of time  -Lack of confidence  -Lack of (communication)skills |
| What prevents you from discussing the patient’s wishes? | - Fear of taking hope - Medical failure - Emotions - Competence - Lack of time |
| What enables you to discuss the patient’s whishes? | - Competencies - Communication skills - In-depth interviewing skills - Sensing the right moment - Effective discussion within the multidisciplinary team - Education and training |
| How do you record/document this information? | - Fixed location - Structure/methods - Easy to find? Also for colleagues? |
| Do you share this information? If so, with whom? | - Colleagues within and outside your institution |
| Which healthcare professionals do you think should have access to this information? | - Why or why not? - Responsibility of the whole team or just the primary physician? |
| Do you think that the patient has an active role in communicating his/her wishes? If so, how do you see that role? | - Initiator  - Preparation |
| When is a conversation about your patient’s preferences and values successful for you? | When would ACP be successful for you? |
| Are you able to obtain funding for ACP conversations? E.g. from health insurers? | - Yes/no |
| What role does that play in whether or not you have these conversations? |  |
| **Summarize the conversation so far and check with the healthcare provider if the summary is correct.** | |
| Are you familiar with the ACP documentation tool within the electronic health record? | - Where to find it - Application - Experiences - Interoperability - Reference work - Front page |
| If familiar, what are your experiences with it? | -It’s use |
| What role does the tool play in discussing and recording your patients’ values, preferences and wishes? |  |
| Should the patient have access to the documentation in the ACP tool? E.g. via the patient portal. |  |
| Should the patient be able to change the documentation themselves? |  |
| With whom/which healthcare professionals should the documentation be shareable? | - With the patient - With colleagues within the institution - With the general practitioner - Others |
| What do you think is needed in your organization encourage you and your colleagues to use the tool (more often)? |  |
| What do you personally need? | - Support tools (e.g. conversation guide) - Training - Education  - Reminders |
| Do you know where to go in your organisation if you have (technical) questions about the tool? |  |
| How convinced are you at this moment to use the tool yourself? And your colleagues? |  |
| Do you think it is feasible for everyone to adopt the tool? | - Why or why not?  - Where should the implementation program focus on? |
| **Closing** | - Thank you for your time and openness - If you wish, I will keep you informed about the research via email. The results will be used for the tailored implementation plan for your institution. |

1. Rietjens JAC, Sudore RL, Connolly M, et al. Definition and recommendations for advance care planning: an international consensus supported by the European Association for Palliative Care. The Lancet Oncology 2017;18:e543-e551.
